# Effect of Plyometric Exercises of Lower Limb on Strength, Postural Control, and Risk of Falling in Stroke Patients

**DOI:** 10.3390/medicina61020223

**Published:** 2025-01-26

**Authors:** Ahmed K. Abd Elsabour, Hoda M. Zakaria, Ebtesam M. Fahmy, Azza Sayed Abdelrehim Khalil, Reem M. Alwhaibi, Walaa M. Ragab, Shreen I. Taha

**Affiliations:** 1 Department of Physical Therapy for Neuromuscular Disorders and Its Surgery, Faculty of Physical Therapy, Beni-Suef University, Beni Suef 62521, Egypt; ahmedkamel@pt.bsu.edu.eg (A.K.A.E.); shreen_taha@pt.bsu.edu.eg (S.I.T.); 2 Department of Physical Therapy for Neurology and Neurosurgery, Faculty of Physical Therapy, Cairo University, Cairo 12613, Egypt; dr.hodazakaria@cu.edu.eg; 3 Department of Neurology, Faculty of Medicine, Cairo University, Cairo 12613, Egypt; em_fahmy@kasralainy.edu.eg; 4 Rehabilitation Sciences Department, Health and Rehabilitation Sciences College, Princess Nourah Bint Abdulrahman University, Riyadh 11671, Saudi Arabia; ASKhalil@pnu.edu.sa (A.S.A.K.); rmalwhaibi@pnu.edu.sa (R.M.A.); 5 Department of Physical Therapy, College of Medical Rehabilitation Sciences, Taibah University, Medina 42353, Saudi Arabia

**Keywords:** stroke, plyometric exercise, muscle strength, balance, fall risk, rehabilitation

## Abstract

*Background and Objective:* Stroke, a major contributor to long-term disability worldwide, often results in significant impairments in motor function. These impairments can include weakness, impaired balance, and decreased coordination, which can have a significant influence on one’s quality of life and independence. Finding an effective protocol for rehabilitation to improve these points will decrease the impact of stroke and its coast of rehabilitation. *Materials and Methods:* This study was conducted to assess the effect of lower limb plyometric exercises on strength, postural control, and risk of falling in stroke patients. *Materials and Methods:* This study involved 40 chronic left stroke patients randomly divided into two equal groups. The experimental group participated in a 12-week supervised plyometric training program, while the control group received conventional physical therapy program. Lower limb muscle strength was measured using a handheld dynamometer, and balance and fall risk were assessed via the Biodex Balance System (BBS). These measurements were conducted before and after the intervention period to evaluate treatment effects. *Results:* The results of this study demonstrated significant improvements in muscle strength and balance parameters among stroke patients who underwent plyometric exercise compared to those receiving a conventional program. The plyometric group exhibited significantly greater increases in knee extension strength (*p* < 0.05), hip abduction strength (*p* < 0.05), ankle dorsiflexion strength (*p* < 0.05), and ankle eversion strength (*p* < 0.05). Furthermore, the plyometric group showed significant improvements in overall stability (*p* < 0.05), mediolateral stability (*p* < 0.05), and anteroposterior stability (*p* < 0.05), as measured by the Biodex Balance System (BBS). *Conclusions:* The results of this study suggest that plyometric exercise may be an effective intervention for decreased risk of falling and enhancing muscle strength and balance during recovery from stroke.

## 1. Introduction

Stroke is a prevalent cerebrovascular condition marked by the rapid development of localized (or widespread) disruption of brain function, persisting for over 24 h or causing death and attributable solely to vascular causes. This condition is linked to high morbidity, mortality, and disability rates, placing a significant economic strain on both society and families [1]. The impairments following a stroke encompass a broad range of clinical signs and symptoms. Disability outcomes, influenced by multiple factors, vary based on the extent of neurological recovery, the location of the lesion, the patient’s prior health status, and the availability of environmental support [2]. In the United States, roughly 795,000 individuals are affected by stroke annually, with many experiencing sensorimotor and cognitive impairments that heavily affect balance control [3].

Postural control, the ability to maintain an upright stance during functional activities while compensating for both external and internal perturbations, is crucial in preventing falls and also affect function of upper limb. Impaired balance, particularly among community-dwelling older women after a stroke, is a significant predictor of falls. Due to compromised balance control, 40% to 70% of stroke survivors experience falls each year, which impedes daily functioning, limits mobility in the community, and can result in long-term disability [4]. The consequences of falls post-stroke can be severe, leading to decreased social participation, fear of falling, traumatic brain injuries, fractures, functional impairments, increased morbidity, and even mortality [5]. Post-stroke, impaired motor control and extremity weakness not only diminish muscular force production but also disrupt the coordination of functional movements between limbs and, thus, affect balance. Muscle strength in the affected lower extremity is typically reduced by 34–62% compared to healthy individuals. Weakness of hip abductors affects the pelvic stability of weight-bearing lower extremity during balance control in medial–lateral directions, leading to difficulty in transferring the body weight towards affected side [6]. Additionally, knee extensor strength indicates overall lower-limb strength in individuals with stroke. Plantar and invertors muscle spasticity impacts dorsiflexion and evertors more significantly, and this effect persists even after the plantar spasticity is resolved, resulting in balance and gait problems [7]. For all these reasons, our study focused on these muscles during the assessment.

Integrating plyometric exercises shows significant promise for enhancing postural control and reducing the adverse impacts of falls, thereby improving functional capacity and overall quality of life in affected persons [8]. Plyometric exercises offer substantial theoretical and practical benefits for training both the upper and lower extremities. These benefits include the potential to improve average power and velocity, increase peak force and acceleration velocity, extend the duration of force development, elevate muscle activation levels, improve cardiovascular fitness, and stimulate stretch reflex responses [9]. Polymetric exercises are commonly used with athletes, and there is limited research on its benefits in neurological cases such as stroke. We performed this study to assess its effect on posture control, falling risk, and strength in patients with stroke.

## 2. Materials and Methods

### 2.1. Subjects

This study group is a prospective, randomized, double-blind, and controlled clinical trial conducted at the outpatient clinic of the Faculty of Physical Therapy, Beni Suef University, from December 2023 to August 2024. It adhered to the Declaration of Helsinki guidelines for human research ethics. Approval was granted by the Institutional Review Board of the Faculty of Physical Therapy, Cairo University (Reference No: P.T.REC/012/004921, Ethical Approval Date: 7 November 2023), and additionally, it was registered at the Pan African Clinical Trial Registry (pactr.samrc.ac.za) database with the number (PACTR202407725870510). The study adhered to the Declaration of Helsinki’s guidelines for the ethical conduct of human studies. All patients received a thorough explanation of all study procedures before signing a written informed consent that acknowledged the security of their data and recognized the study’s nature, purpose, and the opportunity for them to withdraw from it at any point with no fear of implications.

### 2.2. Sample Size

The sample size for this study was calculated prior to its initiation using G*Power software (version 3.1.9.2). The calculation was based on a multivariate analysis of variance (MANOVA) global effect test, with a type I error rate (α) set at 0.05. Effect size estimates were derived from prior studies investigating the impact of plyometric exercises on stroke patients. Sample size calculations revealed that 40 subjects (split equally between groups) would provide 95% power to observe an effect size of 0.95 in primary outcomes, adjusting for an expected 15% loss to follow-up. A convenience sample of 55 patients with unilateral stroke was screened for study eligibility. The study enrolled 40 eligible participants (men and women) from the Faculty of Physical Therapy’s Outpatient Clinic at Beni Suef University. Each participant received detailed information about the study procedures and objectives, including withdrawal rights, and provided informed consent before beginning.

Inclusion criteria included: first-time stroke, so no previous past history of stroke attack; chronic left stroke patients with a stroke duration of more than six months; Modified Ashworth Scale score of 1 or 1+; BMI between 18.5 and 25; age range of 40–65 years; independent ambulation and ability to make a double footed forward jump; and right-handedness. All included patients were physically active before the occurrence of stroke, walking daily for between 30 and 60 min, as is recommended to enhance cardiovascular health, and this was confirmed on the history section of the sheet. Exclusion criteria included any significant neurological conditions, like multiple sclerosis or Parkinson’s disease; severe musculoskeletal impairments or injuries that could compromise safe plyometric exercise performance, such as knee meniscus injuries or lower limb ligament injuries; severe osteoarthritis of the lower extremity joints; or severe cognitive impairments or communication difficulties that would hinder understanding and adherence to instructions during plyometric exercise interventions or assessments. Patients with recurrent stroke history were also excluded. Participants were randomly assigned to either a study or control group (n = 20 per group) by a blinded, independent research assistant using a computer-generated randomization sequence.

### 2.3. Procedure

The study group received a combined intervention comprising a conventional selected physical therapy program as a warming up exercises for ten minutes and plyometric exercises for fifty minutes. The conventional program included stretching of spastic muscles, strengthening of weak muscles, functional mobility exercises, and balance exercises, including static and dynamic balance training with open and closed eyes to increase proprioception and joint stability. The plyometric exercise program was integrated into a motor relearning program guided by the International Classification of Functioning, Disability, and Health (ICF) framework. Such a framework considers how health status, surrounding environmental elements, and individual characteristics work together to shape functional abilities. The plyometric exercises were designed to target specific impairments and included explosive muscle contractions (eccentric-concentric movements). Non-weight-bearing plyometric exercises were implemented in three sets of 10 repetitions per exercise, per session [10]. These exercises targeted four primary muscle groups: knee extensors, hip abductors, ankle dorsiflexors, and foot evertors (Table 1). During these polymetric exercises, the authors ensured that full range of motion was achieved during each exercise and correct posture and technique were maintained, emphasizing a controlled eccentric phase followed by an explosive concentric phase. Regarding rest and recovery, adequate rest periods were allowed between sets and exercises to prevent fatigue and injury.

After establishing a solid foundation with plyometric exercises, the focus shifted towards functional exercises and activities of daily living (ADLs). This approach aligns with motor learning principles, as practicing skills in a context-specific manner can enhance motor relearning and functional abilities. To progressively increase the exercise load, two practice paradigms can be employed: the first is horizontal exercises, which were performed in a supine or prone position, and the second is vertical exercises which were performed in a standing or weight-bearing position. The treatment program conducted involved minimal adaptation to individual needs. Exercises were divided into two one-month blocks, following the guidelines outlined by Elnaggar et al., 2019 (Table 2) [11]. The number of repetitions for the first month was determined based on individual patient performance during the baseline assessment. A one-minute rest period was implemented between exercises. A standard step height of 5 inches was used throughout the intervention.

The control group received an identical conventional physical therapy program to that of the study group. Both groups underwent rehabilitation exercises for eight consecutive weeks (two months), with three sessions per week, and the session duration was one hour for both groups.

Hand-held dynamometry was used to assess muscle strength, and the Biodex Balance System was used to assess postural stability, limits of stability, and fall risk. The Biodex Balance System (BSS) (Biodex Inc., Shirley, NY, USA) is a reliable tool for objectively assessing postural stability. It utilizes a platform that can rotate in various directions, allowing for the measurement of balance parameters. Key assessments include the Mediolateral Stability Index (MLSI), which measures side-to-side stability; the Anteroposterior Stability Index (APSI), which measures front-to-back stability; and the Overall Stability Index (OSI), which provides an overall assessment of balance. Additionally, the Fall Risk Index (FRI), which can also be measured by BBS, can be automatically calculated to quantify the individual’s risk of falling. The FRI provides a quantitative assessment of the subject’s fall risk, with a higher score indicating a higher risk of falling. By providing quantitative data on balance performance, the BSS is priceless for evaluating the effectiveness of rehabilitation interventions and monitoring patient progress [12].

Hand-held dynamometry (HHD) is a practical, objective, and cost-effective tool for quantifying muscle strength. Using a portable device like the Lafayette model 3790, Lafayette instrument company made in Lafayette, IN, USA, examiners used it to assess muscle strength of hip abductors, knee extensors, ankle dorsiflexors and ankle eversion by applying resistance during maximal isometric contractions. This technique is particularly useful for clinical settings, as it allows for quick and reliable strength assessments [13].

### 2.4. Data Processing

Data analysis was completed with SPSS version 25. Descriptive statistics included means and standard deviations. Between-group differences in subject characteristics were assessed via unpaired *t*-tests, and sex distribution was compared using chi-squared analyses. Mixed-model analysis of variance (MANOVA) was conducted to assess both within-group and between-group effects of the treatment on muscle strength, postural stability, and fall risk. Statistical significance was set at a *p*-value of less than 0.05.

## 3. Results

Table 3 provides a detailed summary of the demographic characteristics for both the study and control groups. Notably, the two groups exhibited remarkable similarity in terms of age (*t* test = 2.0865), BMI (*t* test = 0.2699), and sex distribution (qui square test (X^2^ = 0), as there was no significant difference between groups, and this enhances the internal validity of the study.

The provided (Table 4) offers a comprehensive comparison of pre- and post-treatment outcomes for muscle strength, postural stability, and fall risk between the study and control groups. The study group exhibited significantly greater improvements post-treatment compared to the control group in terms of muscle strength, anteroposterior stability, mediolateral stability, overall stability index, and fall risk index (*p* < 0.05).

The provided table (Table 4) offers also a comprehensive comparison within-group analysis. Both the study and control groups displayed significant improvements in muscle strength after treatment (*p* < 0.05), while significant improvements were observed only in anteroposterior stability, mediolateral stability, overall stability index, and fall risk index post-treatment in the study group (*p* < 0.05).

## 4. Discussion

This study significantly advances the understanding of the role of plyometric exercises in stroke rehabilitation, demonstrating their potential to enhance lower limb strength, improve postural control, and reduce fall risk. By comparing the findings with previous research, we gain valuable insights into the efficacy and applicability of plyometric training across diverse populations and contexts.

Falling is a frequent complication following a stroke, with both physical impairments (including weakness, paralysis, sensory disruptions, and compromised postural control) and mental challenges (including mental fatigue, depression, and cognitive impairment) contributing to recurrent falls [14]. Commonly, stroke survivors experience decreased muscle strength in the affected limbs, reduced power generation, muscle weakness, and limited joint range of motion [15]. Research has demonstrated that plyometric exercise can significantly enhance lower limb strength in stroke survivors. These exercises stimulate fast-twitch muscle fibers, leading to muscle growth and neuromuscular adaptations. This increased strength not only facilitates balance and gait restoration but also improves the ease of performing daily activities. Additionally, plyometric training has been shown to enhance postural control, a critical component of movement stability [16].

According to the current study, there was a significant difference in post-treatment mean scores of muscle strength, which aligns with the findings of Luo et al., 2019, who demonstrated that plyometric exercises can significantly increase strength and functional mobility in stroke patients, who often experience muscle weakness and poor motor control [17]. The present study demonstrates a significant increase in lower limb strength among participants who engaged in plyometric training, as evidenced by enhanced performance on strength assessments. These findings align with the results of Elnaggar, 2022, who also observed substantial improvements in muscle strength among stroke patients following a structured plyometric training program [8]. Both studies highlight the potential of plyometric exercises to induce significant neuromuscular adaptations, including increased motor unit recruitment and muscle fiber hypertrophy, which are crucial for improving functional strength in stroke survivors. The current study extends the scope of existing research by observing the impact of plyometric training on muscle endurance and fatigue resistance, critical factors for sustaining physical activity over time. The significant improvements observed in these areas suggest that plyometric exercises may help mitigate the effects of muscle fatigue, a common challenge in stroke rehabilitation. This finding is supported by the work of [18], who noted that stroke patients may experience enhancements in upper and lower extremity motor function through the utilization of high-intensity exercise protocols. Additionally, ref. [19] concluded that supervised plyometric programs can safely replace traditional strength training specifically targeting improvements in dynamic neuromuscular performance.

Improved postural control was another key outcome of our study, with participants showing significant differences in post-treatment mean scores of anteroposterior stability, mediolateral stability, and overall stability, these findings align with those of [10,20], However, our study makes a novel contribution by extending these benefits to a stroke population, where balance impairments are a leading cause of disability and reduced quality of life. Additionally, our study provides evidence that the improvements in postural control are not solely due to strength gains but also to enhanced neuromuscular control, as plyometric exercises stimulate the central nervous system in ways that improve motor planning and execution, thereby enhancing balance and reducing the risk of falls. Thus, polymetric exercises offer a dual benefit of strengthening the musculoskeletal system and refining neuromuscular control. Reducing the risk of falls is a critical goal in stroke rehabilitation, and our study provides strong evidence that plyometric training can contribute to this objective and this outcome aligns with the findings of [21]. Moreover, our study’s findings resonate with the conclusions of Moreland et al., 2004, who emphasized the importance of high-intensity, dynamic exercises in fall prevention programs for older adults. By applying this principle to a stroke population, our research underscores the potential of plyometric training to serve as a key component of multifaceted fall prevention strategies. The decrease in fall risk may be attributed to improvements in both physical and psychological factors, such as increased confidence in balance abilities and reduced fear of falling, which are crucial for encouraging greater physical activity and reducing sedentary behavior among stroke survivors [22].

The results of this study have several important clinical implications. First, they suggest that plyometric exercises could be integrated into standard stroke rehabilitation protocols to enhance strength, balance, and reduce fall risk. Given the observed benefits, clinicians should consider including plyometric training in the rehabilitation programs of stroke survivors, particularly those in chronic phases of recovery.

However, while the results are promising, further research is needed to optimize plyometric training protocols for stroke rehabilitation. For example, determining the optimal intensity, frequency, and duration of plyometric exercises for different stages of stroke recovery or older patients could enhance the effectiveness of these interventions. Moreover, future studies should explore the safety and feasibility of plyometric training in more severe stroke cases, where balance and strength deficits may be more pronounced. Also, future research could include tests like the Timed Up-and-Go Test or the 6-Minute Walk Test, the fatigue scale, and the muscle endurance test to correlate the effect of polymetric training with these variables in patients with stroke.

## 5. Conclusions

In conclusion, our findings add to the accumulating evidence highlighting the benefits of plyometric exercises in the rehabilitation of stroke patients. By improving lower limb strength, postural control, and reducing the risk of falls, plyometric training offers a powerful tool for enhancing functional recovery in stroke survivors. These findings should encourage clinicians to consider integrating plyometric exercises into standard rehabilitation protocols, with the goal of improving long-term outcomes for stroke patients.

## Figures and Tables

**Table 1:** Plyometric exercise protocol.

	Exercise 1: Supine Leg Press (Knee Extensor)	Exercise 2: Clamshells (Hip Abductor)	Exercise 3: Ankle Dorsiflexion	Exercise 4: Foot Eversion
**Eccentric Phase**	Patient lies supine with knees flexed and feet flat on the bed/floor. Therapist applies resistance to knee flexion.	Patient lies on their side with knees bent at a 90-degree angle. Therapist applies resistance to hip adduction.	Patient lies on the back with the knee flexed at a right angle. Therapist applies resistance to plantarflexion.	Patient lies supine with the foot in an everted position. Therapist applies resistance to inversion.
**Concentric** **Phase**	Patient forcefully extends the knee against therapist resistance.	Patient forcefully abducts the top leg against resistance.	Patient forcefully dorsiflexes the ankle against resistance.	Patient forcefully everts the foot against resistance.

**Table 2:** Paradigm exercises.

Horizontal Training Paradigm			
Exercise	Prescription	Repetition in 1st Month	Repetition in 2nd Month
**Forward jumping**	Double-footed forward jump to maximum achievable distance preceded arm swing	1 set/5 R	1 set/10 R
**Walking Sideward**	Practice walking using lateral side-steps	Walk each side 1 min	Practice walking using crossed-steps and side-steps each side for 1 min
**Walking Backward**	Practice walking Backward (retro-walking)	Walking on ground for 1 min	Walking backward over obstacle 1 min
**Vertical training paradigm**			
**Step up**	Stand behind a step, step up and then back down, switching between RT and LT foot each time.	1 set/5 R	1 set/10 R
**Marching on** **a foam pad**	The patient stands on a dense foam pad, The patient performs alternating hip and knee flexion, marching in place. The therapist provides verbal cues and guarding	2 sets/10 R with eye open	2 sets/10 R with eye closed

R: repetitions; RT: right; LT: left.

**Table 3:** General characteristics of participants.

Items	Groups (Mean ± SD)		*p* Value
	Study Group	Control Group	
Age (year)	54.5 ± 4.64	54.4 ± 3.2	0.937 (NS)
BMI (kg/m^2^)	19 ± 1.43	19 ± 2.13	0.78 (NS)
Sex			
Male	12 (60%)	12 (60%)	1
Female	8 (40%)	8 (40%)	

SD: standard deviation, BMI: body mass index, *p*: value probability value, NS: non-significant.

**Table 4:** Comparison of measured variables pre- and post-treatment within and between both groups.

Variables		Groups (Mean ± SD)		*F Value*	*p* Value
		Study Group	Control Group		
Muscle strength					
Knee extensor	Pre-treatment	73.4 ± 8.35	72.5 ± 7.96	1.10	0.72
	Post-treatment	95.9 ± 8.64	83.7 ± 4.71	3.36	<0.0001 (S)
	*F value*	1.07	2.86		
	*p* value	<0.0001 (*S*)	<0.0001 (*S*)		
Hip abductors	Pre-treatment	57.7 ± 7.07	54.9 ± 4.28	2.79	0.13
	Post-treatment	76.1 ± 7.63	65.7 ± 3.91	3.81	<0.0001 (S)
	*F value*	1.16	1.20		
	*p* value	<0.0001 (*S*)	<0.0001 (*S*)		
Ankle dorsiflexion	Pre-treatment	21.7 ± 4.08	23.5 ± 3.34	1.49	0.13
	Post-treatment	35.2 ± 6.42	30.9 ± 4.84	1.76	<0.02 (S)
	*F value*	2.48	2.10		
	*p* value	<0.0001 (*S*)	<0.0001 (*S*)		
Ankle eversion	Pre-treatment	16 ± 2.98	14.8 ± 2.14	1.94	0.15
	Post-treatment	21.7 ± 3.52	17.7 ± 1.8	3.82	<0.0001 (S)
	*F value*	1.40	1.14		
	*p* value	<0.0001 (*S*)	<0.0001 (*S*)		
Postural stability					
Overall stability index	Pre-treatment	3.44 ± 0.52	3.78 ± 0.59	1.29	0.06
	Post-treatment	2.74 ± 0.68	3.46 ± 0.61	1.24	0.0001 (*S*)
	*F value*	1.71	1.07		
	*p* value	0.0008 (*S*)	0.09		
Anteroposterior stability	Pre-treatment	2.24 ± 0.39	2.45 ± 0.31	1.58	0.06
	Post-treatment	1.81 ± 0.47	2.43 ± 0.29	2.63	<0.001 (*S*)
	*F value*	1.45	1.14		
	*p* value	0.003 (*S*)	0.8		
Mediolateral stability	Pre-treatment	2.11 ± 0.46	2.35 ± 0.39	1.39	0.08
	Post-treatment	1.79 ± 0.5	2.16 ± 0.39	1.64	0.01 (*S*)
	*F value*	1.18	1.00		
	*p* value	<0.041 (*S*)	0.13		
Fall risk test					
Overall stability index	Pre-treatment	3.69 ± 0.35	3.95 ± 0.53	2.29	0.7
	Post-treatment	2.47 ± 0.51	3.85 ± 0.54	1.12	<0.001 (*S*)
	*F value*	2.12	1.04		
	*p* value	<0.0001 (*S*)	0.55		

mean ± SD: mean ± standard deviation, (*S*) significant (*p* < 0.05), *p* probability.

## Data Availability

The data can be requested from the corresponding author and will be released upon reasonable request.
